# Advanced gastric cancer with brain metastasis effectively treated by arterial infusion chemotherapy: A case report

**DOI:** 10.3892/ol.2013.1699

**Published:** 2013-11-22

**Authors:** ZHAOHONG PENG, SHENGDE XU, HUA LI, CHAOBIN SUN, MINYAN FU, MINGZHU GAO

**Affiliations:** 1 Department of Interventional Radiology, Bin Hu Hospital of Hefei, Hefei, Anhui 230000, P.R. China; 2 Department of Interventional Radiology, Third Affiliated Hospital of Anhui Medical University, Hefei, Anhui 230000, P.R. China

**Keywords:** advanced gastric cancer, intra-arterial infusion, brain metastasis

## Abstract

The current report presents a case of advanced gastric cancer with brain metastasis effectively treated by intra-left gastric arterial and internal carotid arterial infusions of tegafur, epirubicin and lobaplatin. The patient was a 73-year-old male complaining of headache, nausea/emesis and discomfort in the upper abdomen for six months and was found to have advanced gastric cancer with brain metastasis. The patient was treated by intra-left gastric arterial infusion of 800 mg tegafur, 20 mg epirubicin hydrochloride and 30 mg lobaplatin; and intra-left internal carotid arterial infusion of 400 mg tegafur, 10 mg epirubicin hydrochloride and 20 mg lobaplatin. Following four cycles of intra-arterial infusion chemotherapy, the patient’s brain metastasis and discomfort in the upper abdomen had disappeared. The treatment appeared effective for advanced gastric cancer with brain metastasis. However, further investigation in a large-sample study is required to confirm its validity.

## Introduction

As the fourth most common type of cancer and the second most common cause of cancer-related mortality worldwide, gastric cancer has a high incidence, particularly in Asia (1,2). However, the majority of gastric cancers have progressed to an advanced stage at the time of diagnosis, including liver, lymph node, lung or bone metastasis, or peritoneal dissemination. However, advanced gastric cancer with brain metastasis is extremely rare and results in a poor prognosis (3). The current study presents a case of gastric cancer with brain metastasis effectively treated with arterial infusion by a combination of tegafur, epirubicin and lobaplatin. Written informed consent was obtained from the patient.

## Case report

**Figure 1:**
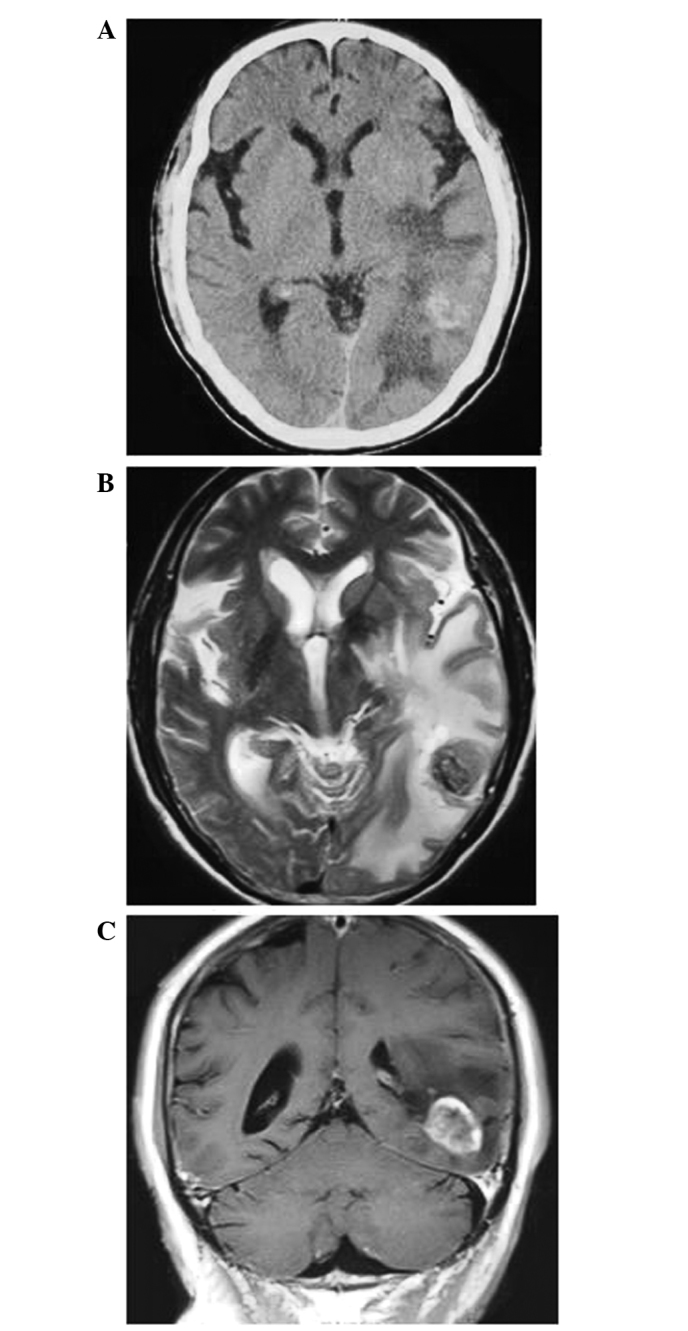
Prior to treatment, (A) axial CT, (B) axial MRI T2-weighted and (C) coronal MRI T1-weighted scans reveal brain metastasis in the left temporal lobe and cerebral edema located around the lesion. CT, computed tomography; MRI, magnetic resonance imaging.

**Figure 2:**
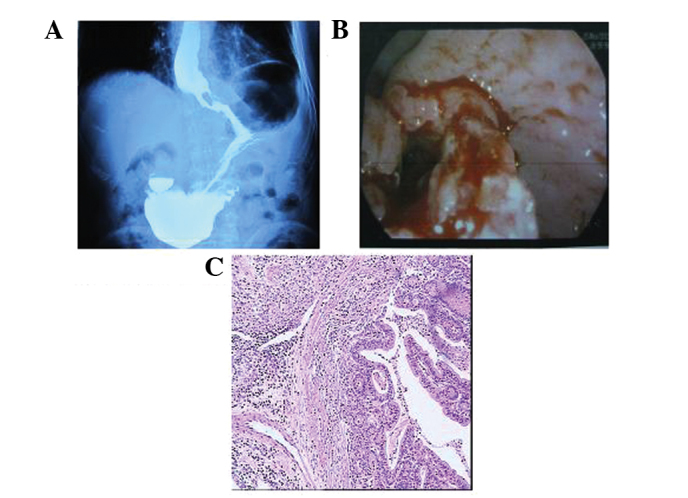
(A) Barium esophagogram and (B) endoscopic examination show the primary lesion located in the gastric cardia. (C) Pathological examination shows a poorly differentiated adenocarcinoma (hematoxylin and eosin; magnification, ×200).

A 73-year-old male was admitted to the Third Affiliated Hospital of Anhui Medical University (Hefei, China) with headache, nausea/emesis and a disturbance in the nervous system (convulsions and balance/gait problems). Computed tomography and magnetic resonance imaging scans revealed a cerebral tumor in the left temporal lobe and cerebral edema was located around the lesion (Fig. 1A–C). Simultaneously, the patient complained of discomfort in the upper abdomen for six months and had experienced a 10-kg weight loss. Therefore, the patient received barium esophagogram and endoscopic examination (Fig. 2A and B). The results demonstrated advanced gastric cancer with a primary lesion located in the gastric cardia. Pathological examination considered the lesion to be a poorly differentiated adenocarcinoma (Fig. 2C). The laboratory examination identified carcinoembryonic antigen (CEA) levels of 283.4 ng/ml and carbohydrate antigen (CA) 19-9 levels of 283.4 U/ml, and the stool was positive for occult blood test (3+). No symptoms of anemia were identified.

**Figure 3:**
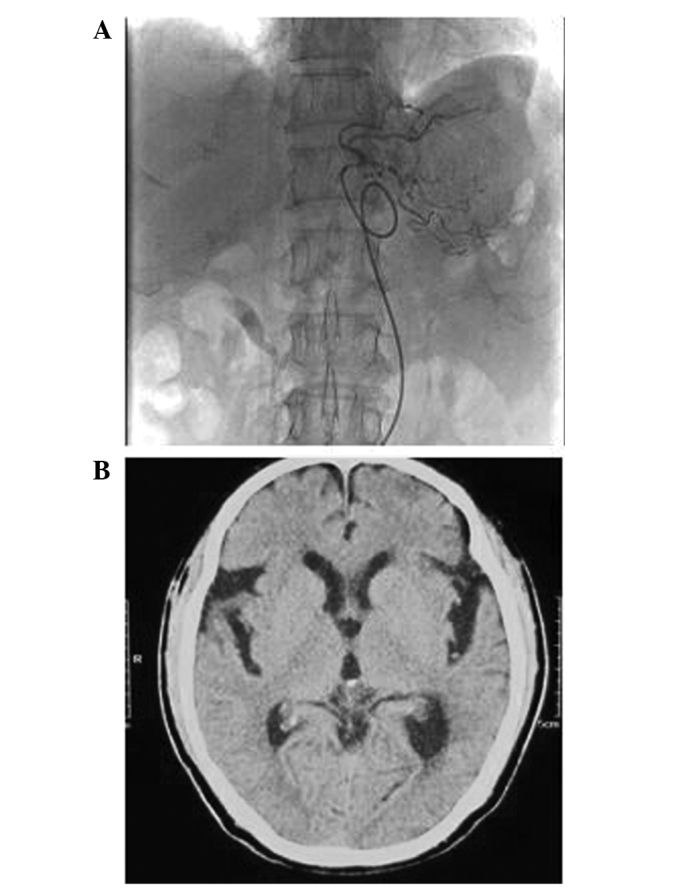
(A) Angiography reveals blood supply to the primary lesion via the left gastric artery. (B) Axial CT scan shows that the brain metastasis in the left temporal lobe has disappeared following four cycles of intra-arterial infusion chemotherapy. CT, computed tomography.

Intra-arterial infusion of anticancer drugs was performed on June 26th, 2012. Angiography revealed blood supply to the primary lesion via the left gastric artery (Fig. 3A) and to the brain metastasis via the left internal carotid artery. Therefore, the method of one-shot intra-arterial injection was adopted. The catheter was inserted into the left gastric artery for infusion of 800 mg tegafur, 20 mg epirubicin hydrochloride and 30 mg lobaplatin; and it was inserted into the left internal carotid artery for infusion of 400 mg tegafur, 10 mg epirubicin hydrochloride and 20 mg lobaplatin. Following four cycles of intra-arterial infusion chemotherapy (received approximately every one month), the metastatic brain tumor (Fig. 3B) and discomfort in the upper abdomen had disappeared. Simultaneously, the neurological symptoms of the patient were completely improved. However, serum CA19-9 and CEA levels (268.7 U/ml and 270.2 ng/ml, respectively) did not markedly decrease.

## Discussion

Gastric cancer is the most common malignant tumor worldwide. In China, gastric cancer is diagnosed in ~400,000 individuals every year, accounting for 42% of the global incidence of gastric cancer. However, gastric cancer with brain metastasis is extremely rare. Previously, York *et al*(4) reported that only 24 out of 3,320 (0.7%) gastric cancer patients were identified with brain metastasis during a 40-year period (1957–1997) at the MD Anderson Cancer Center. In addition, Kasakura *et al*(5) identified brain metastasis in only 11 out of 2,322 (0.47%) patients between 1980 and 1998. The incidence of brain metastases is markedly lower than that of lung, breast, renal and other types of cancer. Associated clinical studies have shown that the majority of the gastric cancer patients with brain metastases exhibit primary lesions located in the gastric cardia. The patient in the current study exhibited a primary lesion also located in the gastric cardia. Advanced gastric cancer patients with brain metastases usually have a poor prognosis and a poor median survival rate (4–7).

Treatment for advanced gastric cancer with brain metastases is usually conservative, palliative and aimed at reducing the patient’s discomfort and improving the quality of life. Traditional treatment methods include systemic chemotherapy, whole brain radiotherapy, γ-knife radiosurgery and palliative surgical resection; however, the improvement in the median overall survival rate has remained dissatisfying (4,8,9). Intra-arterial infusion chemotherapy is performed using a catheter inserted into the artery supplying blood to the tumor. A highly concentrated chemotherapeutic agent is injected directly into the primary lesion and/or metastasis. The advantages of the treatment include delivery of a greatly improved chemotherapeutic agent to the tumor site and a reduction of adverse effects on the various systems of the body (10). In the present case report, the metastatic brain tumor and discomfort in the upper abdomen disappeared following treatment. In addition, the neurological symptoms of the patient were completely improved following four cycles of intra-arterial infusion chemotherapy. Overall, intra-arterial infusion chemotherapy was effective for advanced gastric cancer with brain metastasis in the present case. However further investigation is required in a large-sample study to confirm its validity.
